# The function and therapeutic targeting of anaplastic lymphoma kinase (ALK) in non-small cell lung cancer (NSCLC)

**DOI:** 10.1186/s12943-018-0810-4

**Published:** 2018-02-19

**Authors:** Brandon Golding, Anita Luu, Robert Jones, Alicia M. Viloria-Petit

**Affiliations:** Department of Biomedical Sciences, Ontario Veterinary College, University of Guelph, 50 Stone Road East, Guelph, ON N1G 2W1 Canada

**Keywords:** Lung cancer, Anaplastic lymphoma kinase, ALK, Molecular-targeted therapy, Cell signalling

## Abstract

Lung cancer is the leading cause of death by cancer in North America. A decade ago, genomic rearrangements in the anaplastic lymphoma kinase (ALK) receptor tyrosine kinase were identified in a subset of non-small cell lung carcinoma (NSCLC) patients. Soon after, crizotinib, a small molecule ATP-competitive ALK inhibitor was proven to be more effective than chemotherapy in ALK-positive NSCLC patients. Crizotinib and two other ATP-competitive ALK inhibitors, ceritinib and alectinib, are approved for use as a first-line therapy in these patients, where ALK rearrangement is currently diagnosed by immunohistochemistry and in situ hybridization. The clinical success of these three ALK inhibitors has led to the development of next-generation ALK inhibitors with even greater potency and selectivity. However, patients inevitably develop resistance to ALK inhibitors leading to tumor relapse that commonly manifests in the form of brain metastasis. Several new approaches aim to overcome the various mechanisms of resistance that develop in ALK-positive NSCLC including the knowledge-based alternate and successive use of different ALK inhibitors, as well as combined therapies targeting ALK plus alternative signaling pathways. Key issues to resolve for the optimal implementation of established and emerging treatment modalities for ALK-rearranged NSCLC therapy include the high cost of the targeted inhibitors and the potential of exacerbated toxicities with combination therapies.

## Background

Anaplastic lymphoma kinase (ALK) is a transmembrane receptor tyrosine kinase that belongs to the insulin receptor superfamily [1]. Originally identified as a fusion gene in anaplastic large-cell lymphoma (ALCL), the function of native ALK is not fully understood. Studies on the spatial and temporal expression of ALK in mice have pointed to a role for ALK in fetal nervous system development. By 3 weeks of age, mRNA and protein levels are dramatically reduced and remain low throughout adulthood [2–4]. Interestingly, ALK expression is nearly undetectable in adult mice, and *Alk*-knockout mice are viable, displaying only minor behavioral phenotypes, indicating that ALK is not absolutely required for proper growth and development [5]. The ligand(s) that bind and activate ALK remain a matter of debate. Two of the suspected ALK ligands are pleiotrophin and midkine, as they exhibit a distribution pattern in mice that is similar to that of ALK. [6–8]. While initial studies demonstrated neurotrophic activity of these two growth factors upon receptor binding [6] subsequent reports have failed to detect similar effects [9–11]. More recently, heparin [12] and two members of the family with sequence similarity (FAM), 150A (FAM150A) and 150B (FAM150B) [13, 14], were identified as ALK ligands. In addition to activating wild type ALK, FAM150A/B promote “superactivation” of activated ALK mutants from neuroblastoma [13].

The nucleophosmin (NPM)-ALK fusion gene was the first alteration in the ALK gene to be discovered in human cancers. Characterized by a translocation between chromosomes 2 and 5, the resulting fusion gene leads to constitutive activation of ALK and downstream signaling pathways that drive oncogenesis [1]. Following the discovery of the NPM-ALK fusion gene in ALCL a multitude of different ALK fusion partners have been identified [15, 16]. Three criteria surround the production of oncogenic ALK fusion proteins [17]. Firstly, the breakpoint in the ALK gene occurs such that the entire tyrosine kinase domain is included in the fusion protein (usually at exon 20). Secondly, the promoter region always originates from the fusion partner, presumably due to the fact that the ALK promoter is not active in adults and therefore is not capable of driving transcription of the fusion gene. Finally, the fusion partner must contain an oligomerization domain [17]. Normally, binding of pleiotrophin, midkine, or heparin to the unaltered ALK receptor results in dimerization, transphosphorylation of the tyrosine kinase domains, and subsequent activation [12]; however, the presence of an oligomerization domain in the fusion partners of ALK fusion proteins results in ligand-independent dimerization, and therefore continuous activation of the abnormal receptor [17]. ALK fusions are commonly observed in ALCL and account for 60-80% of ALCL cases [18].

In addition to oncogenic fusion genes, other types of genetic alterations in the ALK gene that promote tumorigenesis have been identified. For example, point mutations and amplifications of ALK have been observed with high prevalence in the childhood cancer neuroblastoma [19, 20]. F1174 L and R1275Q are prominent gain-of-function mutations in the tyrosine kinase domain that are associated with increased expression and kinase activity of ALK [20, 21]. ALK amplifications are also associated with increased protein expression and activity [19].

### ALK and non-small cell lung carcinoma

Lung cancer is the leading cause of cancer deaths in North America, accounting for about 26% of cancer-related deaths in both men and women in Canada [22], and for 27% and 25% of cancer related deaths in men and women, respectively, in the United States [23]. Lung cancer has been historically categorized into two main histological groups: non-small cell lung carcinomas (NSCLCs) and small cell lung carcinoma (SCLC), accounting for 85% and 15% of lung cancers, respectively. However, the 2015 World Health Organization (WHO) classification includes SCLC into the new category of neuroendocrine tumors [24]. NSCLC is further divided into 3 different subgroups: squamous cell carcinoma, adenocarcinoma, and large cell carcinoma. Patients with NSCLC are not usually diagnosed until advanced stages, and median survival time after diagnosis is usually less than 1 year [25].

Mutations in *KRAS* (Kirsten ras sarcoma viral homolog) and *EGFR* (epidermal growth factor receptor) are the two most common genetic events in lung adenocarcinoma and account for 30% and 15% of cases respectively [26]. Notably, activating mutations in *KRAS* and *EGFR* occur in a mutually exclusive manner and thus represent distinct subgroups of the disease. While therapeutic targeting of mutant *KRAS* remains a significant challenge, the successful use of tyrosine kinase inhibitors (TKIs) for the treatment of patients with EGFR mutant tumors has dramatically altered the management and direction of lung cancer treatment. Indeed, the clinical efficacy and experience with EGFR inhibitors led to the rapid implementation of ALK inhibitors for the treatment of patients with ALK-positive tumors.

**Fig. 1:**
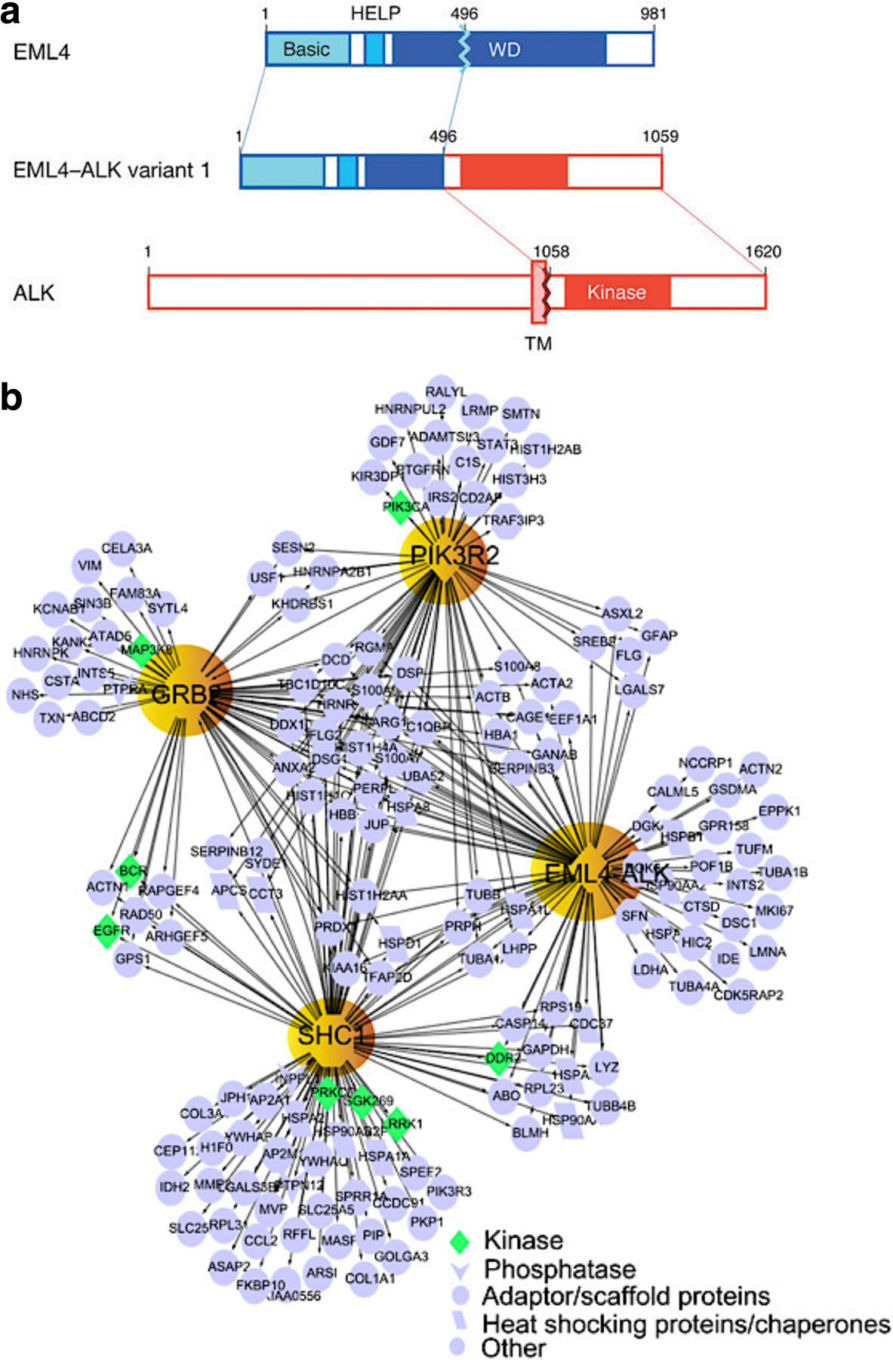
EML4-ALK fusion and its signaling network. **a** Diagram shows the fusion of the N-terminal portion of EML4, which contains its basic region, the echinoderm microtubule-associated protein-like protein (HELP) domain, and part of the WD-repeat region, to the intracellular region of ALK, containing the tyrosine kinase domain. The transmembrane (TM) domain is not present in the final fusion product. Reproduced from ref. [25]. **b** EML4-ALK protein complex network (interactome) constructed using a tandem affinity purification approach followed by mass spectrometry. Reproduced from ref. [39]

In 2007, Soda et al. discovered the echinoderm microtubule-associated protein-like 4 (EML4)-ALK fusion gene (Fig. 1a) in a subset of NSCLC patients. This fusion is the result of an inversion at the short arm of chromosome 2, where the EML4 and ALK genes are located in humans [25]. Following the same criteria described above, EML4 contains a coiled-coil oligomerization domain, which mediates dimerization and constitutive activation of ALK. Like in ALCL, many different ALK fusion partners have been discovered, but EML4-ALK is the most common variant [17]. *ALK* rearrangements are responsible for 3-7% of NSCLCs, predominantly of the adenocarcinoma subtype and occur in a mutually exclusive manner with *KRAS* and *EGFR* mutations [27]. Although they represent a small proportion of NSCLC cases, the absolute number of ALK-positive NSCLC patients is greater than that of ALK-positive ALCL due to the greater worldwide incidence of lung cancer [17]. Interestingly, ALK-positive NSCLC patients are usually younger and light or non-smokers [28].

Direct proof of the oncogenic potential of EML4-ALK in lung cancer pathogenesis has been demonstrated in mice. Transgenic overexpression of EML4-ALK in type-II alveolar cells of the lung via the surfactant protein-c (SPC) or Clara cell secretory protein (CCSP) promoter led to the rapid development of tumors with features of lung adenocarcinoma [29, 30]. In addition, a recent study by Maddalo et al. utilized CRISPR/Cas9 (clustered regularly interspaced short palindromic repeats/ CRISPR-associated protein 9) gene editing to induce an EML4-ALK rearrangement in vivo that also resulted in lung tumor initiation [31]. Importantly, these models also displayed sensitivity to ALK inhibition and thus serve as valuable tools to explore the mechanisms of EML4-ALK induced lung cancer and response to ALK targeted therapies.

#### Oncogenic activation of signaling pathways by altered ALK

Identification of the signaling networks mediated by ALK is critical to our understanding of the biology of ALK-driven tumorigenesis and the development of effective therapies. This is complicated by the various alterations in ALK that are found in human cancers including fusions, point mutations and amplifications. Much of our understanding of the pathways activated by ALK has come from in vitro studies utilizing NPM-ALK and EML4-ALK based model systems [32]. Signals initiated by constitutively active ALK fusion genes are transmitted through direct interaction of the intracellular kinase domain with various signaling molecules including protein kinases and adaptor proteins with specific interactions likely dictated by the cytoplasmic location of the fusion gene [28]. The JAK-STAT (Janus kinase - signal transducers and activators of transcription) [33], MAPK/ERK (mitogen activated protein kinase/extracellular signaling regulated kinase) [34], PLCγ (phospholipase C gamma) and PI3K-AKT (phosphatidylinositol-3-kinase – AKR mouse thymoma) [35] pathways are four key signaling pathways implicated in mediating the oncogenic effects of deregulated ALK activity. All of these pathways are known regulators of cell cycle progression, proliferation, and apoptosis/cell survival, and their dysregulation is a common feature of human cancers [17]. With regards to lung cancer, the H2228 and H3122 human lung cancer cell lines are EML4-ALK-positive (though they carry different variants) and have been widely used to dissect ALK signaling. Elevated levels of phosphorylated AKT, ERK and STAT3 have been observed in both cell lines, but ALK inhibition results in differential effects on the activation status of these signaling molecules [36]. This suggests the impact of ALK inhibitors on downstream signaling is dependent on the nature of the fusion protein. The importance of PI3K-AKT signaling in EML4-ALK rearranged lung cancer is uncertain as other studies observed activated ERK and STAT3 but not AKT in the same cell lines [37, 38]. Recently, a more comprehensive view of EML4-ALK signaling in lung cancer was revealed using a combination of phosphoproteomics, tandem-affinity precipitation and RNAi [39]. In addition to identifying important roles for molecules known to interact with ALK such as the adaptor proteins GRB2 (growth factor receptor-bound protein 2) and SHC1 (Src homology 2 domain-containing transforming protein 1), numerous kinases, phosphatases and scaffolding proteins were identified that play a critical role in mediating survival of EML4-ALK positive cells. This vast knowledge base of the EM4L-ALK signaling network (Fig. 1b) in lung cancer cells represents an invaluable resource for the identification of potential targets for ALK combination therapy.

### Diagnostic methods for ALK-rearranged NSCLC

#### Fluorescence in situ hybridization

**Fig. 2:**
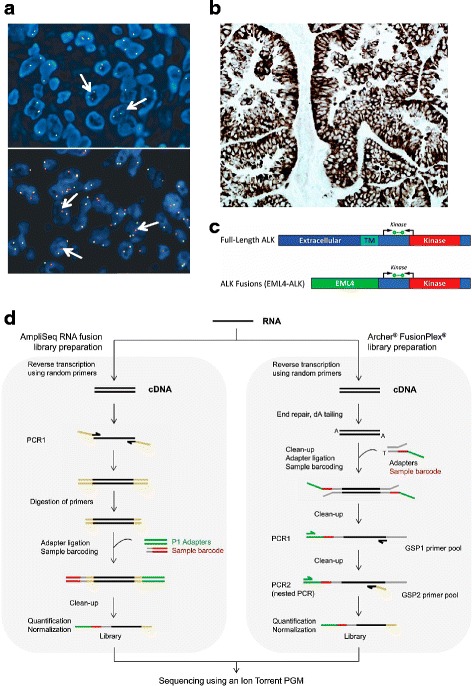
Diagnostic methods for the detection of ALK rearrangement and expression in NSCLC. **a** FISH: arrows in the upper picture exemplify the split signal pattern, while the ones in the bottom picture specified the single red signal pattern. **b** IHC using the D5F3 ALK assay. **c** Diagrammatic representation of full length ALK and the EML4-ALK fusion transcripts indicating ALK domains in the ALK protein, location of ALK RT-PCR primers (black arrows) and the fluorescent probe (green bar) used in the ALK RGQ RT-PCR Kit (Qiagen). TM: transmembrane. **d** Comparison of two commercially available methods to generate libraries for NGS. **a** and **b** adapted from ref. [45]. **c** reproduced from ref. [42]. **d** reproduced from ref. [46]

The first (and currently used) FDA-approved detection method for ALK-positive NSCLC was the Vysis Dual Color break-apart fluorescence in situ hybridization (FISH) (Abbot Molecular, Des Plaines, IL) [40]. A green probe is designed to hybridize to the region immediately 5′ to the ALK gene and a red probe hybridizes to the region immediately 3′ [41]. The test is considered positive if more than 15% of tumor cells in a biopsy sample harbor red and green signals that are split by more than two signal diameters, or if they harbor a single, isolated red signal [41] (Fig. 2a). This is a very sensitive method for detecting disruptions in the ALK locus, but given that EML4 and ALK are only separated by 12.5 megabases on chromosome 2p, it can be prone to false negatives when used to detect this particular rearrangement [40]. Furthermore, FISH can only be used to determine whether there is a break in the ALK locus; it cannot be used to distinguish between the different ALK fusion partners [40]. Other disadvantages of FISH include its high cost, the need for specific expertise to interpret the results, and the long turnaround time. Despite these disadvantages, FISH is still the gold standard for the detection of ALK rearrangements and is used as a comparator for validation of other ALK detection methods [42].

#### Immunohistochemistry

The current standard for diagnosing ALK-positive ALCL is the detection of ALK protein expression via immunohistochemistry (IHC) [17]. Using the same antibodies to detect ALK-positive NSCLC yields poor results, likely due to the lower ALK expression in NSCLC [17, 28]. However, highly sensitive ALK antibodies can be fairly reliable in detecting ALK-positive NSCLC [43, 44]. The principle of using IHC in NSCLC diagnosis is based on the fact that normal lung tissue does not express detectable levels of ALK, but NSCLC with rearranged ALK expresses ALK at modest levels [45]. In comparison to FISH, IHC is a cheaper method that requires less expertise, is more commonly available in hospital settings [18, 40], and yields results more quickly than FISH and other tests. However, in some cases, NSCLCs that tested negative for ALK by IHC were reported to be positive by FISH [45] and similar to FISH, IHC does not permit identification of the fusion partner [46]. The IHC test approved by the United States Federal Drug Administration (FDA) for ALK testing is the VENTANA ALK (D5F3) CDx Assay (Ventana Medical Systems, Tucson, AZ, US), intended for qualitative detection of ALK in formalin-fixed paraffin embedded (FFPE) NSCLC tissue (Fig. 2b) stained using a BenchMark XT or BenchMark ULTRA automated staining instrument. Because of this test’s validation in two widely known clinical trials with ALK inhibitors, and the above-mentioned advantages of IHC over FISH, ALK IHC has been promoted as the primary diagnostic test for NSCLC. However, due to the possibility of a false negative with IHC, most laboratories with extensive experience in NSCLC and ALK testing recommend IHC first, followed by confirmation by FISH [45].

#### Reverse transcription PCR

Different ALK fusion partners may result in different dimerization and signaling potentials and thus different tumor biology as well [32]. Therefore, identification of the specific fusion partner can be important when choosing the most appropriate treatment. Reverse transcription-polymerase chain reaction (RT-PCR) can be used to identify the fusion partner, using primers that are specific to known ALK fusion partners. One initial disadvantage of this technique was that many different primers needed to be used before successfully identifying the ALK fusion partner variant, and unknown fusion variants could not be detected [18, 27]. However, more recently developed assays, such as the ALK RGQ RT-PCR Kit (Qiagen, Manchester, UK), address this problem. This is a one-step quantitative RT-PCR (qRT-PCR) assay that detects the expression of mRNA encoding the ALK tyrosine kinase domain after qualification by an endogenous control reaction (Fig. 2c) and permits the identification of mRNA produced by all ALK rearrangements regardless of the fusion partner or variant [42]. In a study comparing the ALK RGQ RT-PCR assay to FISH and IHC using FFPE specimens in an enriched 95 patients cohort, the qRT-PCR identified 100% of the cases (21 patients) with ALK rearrangement determined by FISH, as well as discordant cases that were ALK-negative by FISH and IHC, which were later verified by next generation sequencing [42]. This, together with additional advantages of qRT-PCR, such as rapid turnaround time, ease of analysis, and the use of biopsy or cytology specimens with a smaller tumor content than that needed for accurate FISH and IHC [42], suggest the feasibility of incorporating qRT-PCR into routine ALK diagnosis in NSCLC.

#### Next generation sequencing

The development of molecular approaches for the detection of ALK fusions, such as qRT-PCR can strengthen the accuracy of the diagnosis by resolving discordant or borderline cases. However, one of the main limitations for clinical application is that this method easily highlights known fusions, but may fail to detect new variants and fusion partners due to the low precision of the 3′/5′ imbalance value leading to misdiagnoses [46]. Amplicon-based next generation sequencing (NGS) is an alternative approach to overcome this problem. The two main commercially available amplicon-based methods are the Ion AmpliSeq RNA Lung Cancer Research Fusion Panel (Thermo Fisher Scientific, Waltham, MA, USA) and the Archer® FusionPlex® ALK, RET, ROS1 v2 kit (ArcherDX, Boulder, CO, USA) (Fig. 2d). A recent study comparing these kits to IHC and FISH in a subset of 37 patients with NSCLC, found that the Archer® FusionPlex® kit accurately classified all samples, and permitted the correct identification of one rare DCTN1 (dynactin subunit 1)-ALK fusion, one novel CLIP1 (CAP-GLY domain-containing linker protein 1)-ALK fusion, and one novel GCC2 (GRIP and coiled-coil domain-containing protein 2)-ALK transcript. Interestingly, two out of three patients harboring these rare and novel rearrangements were treated with and sensitive to crizotinib [46]. The Archer® FusionPlex® kit is an easy-to-use laboratory test with kits developed for both PGM sequencer (Thermo Fisher Scientific) and MiSeq sequencer (Illumina) technologies, with a workflow designed to obtain a result in 5 days [46]. This suggests that Archer®FusionPlex® may provide an accurate, effective alternative to FISH testing for the detection of known and new ALK fusions to guide NSCLC diagnosis and therapy.

### Targeted therapy: ALK inhibitors

#### Crizotinib

**Fig. 3:**
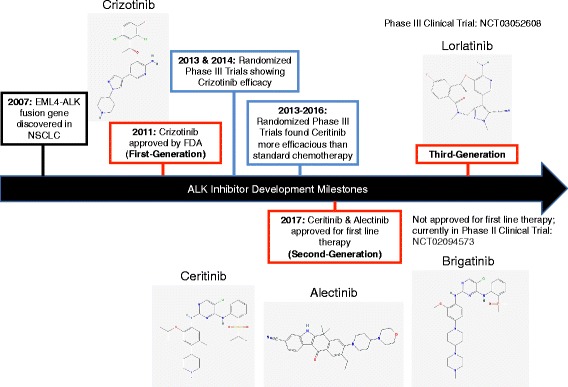
Timeline of ALK Inhibitor Development in NSCLC. EML4-ALK discovery in NSCLC cancer led to the development of first-generation inhibitor crizotinib in 2007. Phase III clinical trials in 2013 and 2014 demonstrated that crizotinib was effective as first line therapy. Due to drug resistance to crizotinib, second-generation inhibitors ceritinib, alectinib and brigatinib were developed. Third-generation inhibitor loratinib is currently in phase III clinical trials. Figure was based on information in references [30, 34, 47, 48, 64, 65, 83]. Chemical structures for the following ALK TKI: crizotinib, ceritinib, alectinib, brigatinib, and lorlatinib were obtained from PubChem [100–104]

In 2011, and only 4 years after Soda et al. discovered ALK-rearrangement as a potential oncogenic driver in NSCLC, crizotinib was approved by the FDA for treatment of advanced ALK-positive NSCLC. Crizotinib is an orally available, small molecule ATP-competitive ALK inhibitor that was originally intended as a MET TKI [34] and then quickly redirected towards ALK upon discovery of the role of ALK rearrangements in NSCLC [17]. A time line of the development of first-, second-, and third-generation ALK TKI discussed in this section is presented in Fig. 3.

#### Crizotinib vs. chemotherapy

Two randomized phase III trials comparing the efficacy of crizotinib to that of second [47] or first-line chemotherapy [48] were reported in 2013 and 2014, respectively. In the first study, 347 patients who presented with ALK-positive lung cancer and had previously received a platinum-based chemotherapy treatment regimen were randomly assigned to receive either oral crizotinib or intravenous chemotherapy with pemetrexed or docetaxel. The study showed a progression-free survival (PFS) of 7.7 months in patients treated with crizotinib compared to 3.0 months in those treated with chemotherapy. A higher objective response rate (ORR) was also observed in crizotinib-treated patients (65% vs. 20%) [47]. The second study enrolled 343 patients who presented with ALK-positive lung cancer but had not previously received any systemic treatment for advanced disease. The patients were randomly assigned to receive either oral crizotinib or intravenous platinum-based double-agent chemotherapy (pemetrexed plus either cisplatin or carboplatin). Similar to the first study, an improved PFS was seen in the patients receiving crizotinib (10.9 vs 7.0 months), as well as a higher ORR (74% vs 45%) [48]. Neither study showed a significant difference in overall survival (OS) of patients between the two treatment groups. One possible explanation is the considerable crossover of patients from the chemotherapy to the crizotinib treatment group upon disease progression. Patients in both studies reported greater reductions in symptoms of lung cancer and an overall greater improvement in quality of life with crizotinib treatment versus chemotherapy.

#### Resistance to crizotinib

**Fig. 4:**
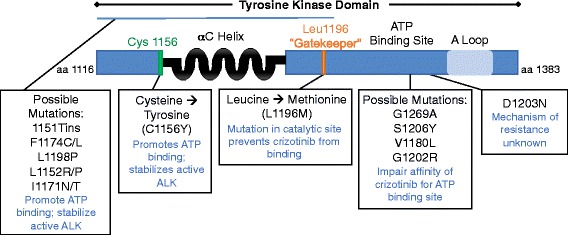
Examples of known mutations in the tyrosine kinase domain of ALK and their influence on kinase activity and drug response. Schematic diagram of the tyrosine kinase domain of the ALK receptor with the location of known mutations. The mechanisms discussed in this review that promote kinase activity and resistance, if known, are indicated. Figure was based on information in references [49–58, 71, 72, 83]

The rapid development of resistance within 1 to 2 years of treatment is a major limitation associated with crizotinib [49]. Mutations in the ALK tyrosine kinase domain are responsible for approximately one third of crizotinib-resistant tumors [50, 51]. The first of these mutations to be discovered were the L1196M and C1156Y mutations [52]. Leucine 1196 is termed the ‘gatekeeper’ residue, as it controls the access of small molecule ALK inhibitors to a hydrophobic pocket within the catalytic site [53]. When this residue is replaced with methionine, or any other amino acid with a bulkier side-chain, it sterically hinders the binding of inhibitors [53]. Numerous variants that confer resistance to crizotinib by impairing its affinity for the ATP-binding site of the kinase domain have since been discovered, including G1269A [49], S1206Y [51], V1180L [54], and G1202R [51]. C1156Y, on the other hand, is predicted to confer resistance through a different mechanism. Being in close proximity to the catalytically important αC-helix within the ALK tyrosine kinase domain, the substitution of cysteine to tyrosine is believed to promote ATP-binding and/or deter inhibitor binding by stabilizing the active confirmation of ALK [49]. Other resistance mutations that map to the same region, and are therefore believed to employ the same mechanism of resistance, are 1151Tins, F1174C/L, L1198P, L1152R/P [49, 55, 56], and I1171N/T [54, 57, 58]. Finally, D1203N is a mutation that occurs at the rim of the ATP-binding site, though the mechanism by which it confers resistance to crizotinib has yet to be determined [55]. Of the mutations conferring resistance to crizotinib, L1196M is the most common, followed by G1269A [49]. A diagrammatic representation of ALK tyrosine kinase domain with the mutations discussed above and how they affect crizotinib activity is presented in Fig. 4.

ALK gene amplification is another potential mechanism of resistance, which is sometimes seen in combination with mutations in the ALK tyrosine kinase domain [50, 51]. Activation of bypass pathways via amplification or mutation of other receptor tyrosine kinases represents another class of resistance mechanism [50, 51]. For example, acquisition of the L858R activating mutation in *EGFR*, results in ALK-independent, aberrant activation of downstream pathways like MAPK or PI3K-AKT, and is frequently observed in crizotinib-resistant tumors [50]. Increased activation of other HER family members beyond EGFR, including HER2 and HER3 may also mediate acquired resistance to crizotinib [59]. In addition, upregulation of IGF1R signaling has been recently identified as an important bypass pathway, and blockade of IGF1R activity resensitized crizotinib resistant cells to ALK inhibition in pre-clinical models [60, 61]. Finally, amplification of *KIT* also represents a potential mechanism of crizotinib resistance, though increased expression of *KIT* alone does not appear to be sufficient to confer resistance [51]. Instead, elevated levels of stem-cell factor (SCF), the ligand for KIT, in the surrounding tumor stroma appears to be required to bypass inhibition of ALK signaling. In some patients, various combinations of these resistance mechanisms have even been detected simultaneously [50].

#### Ceritinib and alectinib

Ceritinib and alectinib are two second-generation ALK inhibitors with acceptable safety profiles that have proven to be effective against many of the prominent forms of crizotinib-resistant ALK-positive NSCLC, including tumors harboring the L1196M gatekeeper mutation [49, 62, 63].

In vitro enzymatic assays have demonstrated the potency of ceritinib to be 20 times greater than that of crizotinib in ALK inhibition, and in vivo studies using the H2228 ALK-rearranged xenograft model revealed that ceritinib has greater efficacy than crizotinib [49]. In phase I and II clinical trials, ceritinib elicited responses in both crizotinib-naïve and crizotinib-refractory patients, independent of whether or not the NSCLC in these patients harbored an ALK resistance mutation. Due to these results, ceritinib was the first ALK inhibitor approved for the treatment of crizotinib-refractory, ALK-rearranged NSCLC [64]. The randomized phase III trials ASCEND-4 and ASCEND-5 found ceritinib to be more efficacious than standard chemotherapy as both first- and second-line therapy [64]. Based on the results of the ASCEND-4 trial, ceritinib was also approved for first-line NSCLC therapy in May 2017. Ceritinib is currently administered at 750 mg daily to fasted patients. However, the recently reported randomized phase I trial ASCEND-8 found that a reduced dose of 450 mg with a low-fat meal has similar effects with improved gastrointestinal tolerability [65].

The second-generation ALK inhibitor alectinib has advantages over both crizotinib and ceritinib, partly due to the fact that it crosses the blood-brain barrier in appreciable quantities [62]. Crizotinib and ceritinib are both targets of p-glycoprotein (P-gp), a membrane protein that pumps xenobiotics out of the central nervous system (CNS), whereas alectinib is not [17]. For this reason, the brain is a common site of relapse in patients treated with crizotinib [62], and alectinib is the best candidate for patients with CNS metastases. A review that compiled 7 trials assessing alectinib in patients with ALK-positive NSCLC that progressed on, were refractory to, or intolerant to crizotinib, including AF-002JG, NP28763 and NP28761, showed that alectinib was highly effective for CNS lesions [66]. A more recent analysis of the pooled results of NP28763 and NP28761 confirmed the promising efficacy of alectinib in the CNS for ALK-positive NSCLC patients pre-treated with crizotinib, regardless of the assessment criteria used [67].

Besides the improved profile of alectinib for the treatment of brain metastasis, the results from a recently published randomized phase III trial comparing alectinib (600 mg twice daily) to crizotinib (250 mg twice daily) in 303 patients with previously untreated, advanced ALK-positive NSCLC (NCT02075840: ALEX) found alectinib to be superior to crizotinib, with a 12-month event-free survival rate of 68.4% for alectinib, as compared to 48% for crizotinib. In addition, 12% of the patients in the alectinib group had an event of CNS progression, as compared with 45% in the crizotinib group (*P* < 0.001). Finally, a response occurred in 82.9% of patients in the alectinib group, compared to 75.5% of the patients in the crizotinib group (*P* = 0.09). Grade 3 to 5 adverse events were also less frequent with alectinib vs. crizotinib (41% vs. 50%) [68].

Based on favorable patient outcomes discussed above alectinib received accelerated approval in December 2015 for the treatment of metastatic ALK-positive NSCLC in patients whose disease progressed on, or were intolerant to crizotinib. In November 2017, alectinib was approved as a first-line therapy for patients with ALK-positive NSCLC at the recommended dose of 600 mg twice daily [69].

#### Sensitivity and resistance to ceritinib and alectinib

As mentioned, both ceritinib and alectinib have proven efficacy against the L1196M gatekeeper mutation. Ceritinib also overcomes other prominent mutations that confer resistance to crizotinib, including G1269A and S1206Y [49], and has also shown activity against I1171T/N in patients and V1180L in Ba/F3 models, both of which confer resistance to crizotinib and alectinib [54, 58, 70]. Alectinib, in turn, has shown activity against C1156Y and F1174C/L in vitro [71, 72], which confer resistance or insensitivity to both crizotinib and ceritinib [51, 71], and against the G1269A variant [72]. As with crizotinib, patients eventually develop a resistance to ceritinib and alectinib [17, 73]. L1152R and 1151Tins are noteworthy, as they conferred resistance to both crizotinib and certinib in Ba/F3 models [49]. Lastly, the ALK G1202R mutation is one that confers resistance to crizotinib, ceritinib, and alectinib [49, 51, 74]. Indeed, in a study performed by Gainor et al. [71], where 103 repeat biopsies from ALK-positive patients progressing on first- and second-generation ALK inhibitors were analyzed, G1202R was the most common resistance mutation identified in the patients receiving second-generation ALK inhibitors. Interestingly, of the patients progressing on the second-generation ALK inhibitors (ceritinib, alectinib, and brigatinib), 56% harbored ALK resistance mutations (*n* = 48), compared to only 20% of those progressing on crizotinib (*n* = 55). Altogether, these data suggest that treatment with second-generation ALK inhibitors is associated with a greater likelihood of developing (or selecting for) resistance mutations, with G1202R being the most common. G1202 is located in the solvent-exposed region of the ALK kinase domain, and substitution of arginine at this location likely leads to steric hindrance of ALK inhibitors due to the larger, charged side chain [51].

As with crizotinib, activation of bypass pathways has been observed in patients and pre-clinical models that are resistant to ceritinib and alectinib. However, ALK resistance mutations are likely responsible for the majority of cases of resistance to second-generation ALK inhibitors [71]. Moreover, the variety of potential bypass pathways, which are not often identified at appreciable frequencies within cohorts studied, and which are often identified in patients who harbor concomitant ALK resistance mutations, have made it difficult to discern their role in driving resistance to ALK inhibitors. Nonetheless, *MET* amplification has been identified in tumor samples derived from a patient who progressed on ceritinib as well as a patient who progressed on alectinib [75, 76]. Of note is the fact that the patient who progressed on alectinib then had a positive response to crizotinib, which was originally designed as a MET inhibitor. In another study, upregulation of neuregulin-1 (NRG1) conferred resistance to ceritinib, alectinib, and brigatinib (discussed below) in NCI-H3122 cells through activation of EGFR family pathways via the NRG1-HER3-EGFR axis [77]. Consequently, a combination of the EGFR inhibitor afatinib with either alectinib or ceritinib effectively targeted resistant cells [77]. Also of interest, in the study by Gainor et al. [71] *TP53* mutations were identified in 2 post-ceritinib samples and 7 post-alectinib samples out of a total 27 samples analyzed. Alterations in the p53 signaling pathway are amongst the most frequently observed in human cancers [78]. However, no further information was provided on these specimens or the role of *TP53* alteration in conferring resistance to ceritinib and alectinib. Other pathways implicated in resistance to second-generation ALK inhibitors are the SRC, MAPK and PI3K pathways, but further study is required in order to elucidate their exact roles [79].

Two other noteworthy implicated mechanisms of resistance that do not involve activation of bypass pathways are P-gp overexpression and epithelial-to-mesenchymal transition (EMT). As mentioned, crizotinib and ceritinib, but not alectinib, are pumped out of the CNS by P-gp. This is further evidenced by the fact that overexpression of P-gp confers resistance to crizotinib and ceritinib, but not alectinib, and cells are re-sensitized by treatment with P-gp inhibitors [80]. Lastly, EMT has been observed in both pre-clinical and clinical ALK inhibitor-resistant specimens [71, 81]. However, one of these studies demonstrated in vitro that EMT alone does not drive resistance to ALK inhibitors [81].

#### Brigatinib

Brigatinib is another second-generation ALK inhibitor that is not yet approved for first-line treatment, but was reported to overcome resistance to other first and second-generation ALK inhibitors in pre-clinical models [82, 83], and to crizotinib in a randomized, multicenter, phase I/II clinical trial (the ALTA/NCT02094573 trial) [84]. In this trial, the best response to brigatinib with an acceptable safety profile was achieved at a dose of 180 mg per day with a 7-day lead-in at 90 mg daily. This dose caused an overall response rate of 54%, including 4 complete responses, and an intracranial overall response rate of 67% (12 out of 18 patients) in evaluable patients with brain metastases [84]. With the FDA approval of brigatinib for the treatment of crizotinib-resistant, ALK-positive NSCLC (with orphan drug designation for ALK+ NSCLC) in April 2017, there are now 4 drugs available for the treatment of ALK-positive NSCLC. However, the optimal sequence to use them to maximize both quality of life and overall survival of patients is still unclear [85]. So far, only crizotinib, ceritinib, and alectinib are approved for first-line therapy, but the results from an ongoing clinical trial comparing brigatinib to crizotininb in ALK inhibitor naïve patients (the ALTA-1L trial) should indicate whether or not brigatinib could also be recommended for first-line therapy, and will possibly suggest better sequential treatments with these approved drugs [85].

#### Sensitivity and resistance to brigatinib

Brigatinib demonstrated superior inhibition and greater selectivity in vitro for nearly all ALK variants discussed above, including C1156Y, F1174C/L, L1152R and 1151Tins, which are implicated in resistance to crizotinib and ceritinib, I1171N and V1180L, which are implicated in resistance to crizotinib and alectinib, and G1202R, which is implicated in resistance to crizotinib, ceritinib and alectinib [83]. However, as mentioned, the obstinate G1202R resistance mutation has been observed in patients progressing on brigatinib, and it is also the ALK variant that brigatinib inhibits least potently [71, 83]. Still, it is worth noting that brigatinib has greater activity against ALK G1202R than crizotinib or any of the other second-generation ALK inhibitors [83].

#### Third-generation ALK inhibitors

Lorlatinib is an ALK/ROS1 inhibitor currently under testing in phase II and III clinical trials (NCT01970865 and NCT03052608), and has shown promising results with regard to resistance. Lorlatinib overcomes the G1202R mutation and inhibits ALK more potently than brigatinib in Ba/F3 cells [71]. In addition, the presence of ALK resistance mutations predicted sensitivity to lorlatinib in ceritinib-resistant, patient-derived cell lines [71]. Further, lorlatinib may resensitize NCSLC to crizotininb. In a study by Shaw et al. [86] lorlatinib was used to treat a patient with crizotinib-resistant C1156Y ALK-positive NSCLC. Upon relapse on lorlatinib, a biopsy revealed that the tumor had an ALK L1198F mutation, in addition to C1166Y. Interestingly, the L1198F mutation made crizotinib once again effective by enhancing its binding to ALK, even with the original crizotinib-resistant mutation (C1156Y) present [86]. Lorlatinib was also reported to cause complete remission of intrathecal metastasis in a heavily pre-treated ALK-positive lung cancer patient, who experienced progression first after chemotherapy plus crizotinib, and second during alectinib treatment [87]. Together, the above findings indicate the potential for an effective, personalized regimen involving a rotation between first, second and third-generation ALK inhibitors in order to maximize response of ALK-positive NSCLCs. Table 1 summarizes known ALK mutations and their influence on resistance or sensitivity to the ALK inhibitors discussed above. A comprehensive review by Lin et al. [73] can be consulted for additional information on lorlatinib and other ALK inhibitors in clinical trials that are not yet approved by the FDA, such as entrectinib and ensartinib.

**Table 1:** ALK inhibitors discussed and their activity against various ALK resistance mutations

	Drug				
ALK Resistance Mutations	Crizontinib	Ceritinib	Alectinib	Brigatinib	Lorlatinib
L1196M	♦	•	•	•	
G1269A	♦	•	•	•	
S1206Y	♦	•		•	
V1180L	♦	•	♦	•	
G1202R	♦	♦	♦	♦	•
C1156Y	♦	♦	•	•	
1151Tins	♦	♦		•	
F1174C/L	♦	♦	•	•	
L1152R/P	♦	♦ (L1152R)		•	
L1198P	♦			• (L1198F)	
I1171N/T	♦	•	♦	• (I1171N)	
D1203N	♦			•	

♦ Mutation confers resistance/insensitivity to the inhibitor

• Inhibitor overcomes resistance mutation

### Future directions

#### Sequential therapy with ALK inhibitors

As discussed above, one strategy to improve the outcome of ALK-positive NSCLC patients under consideration is the sequential treatment with different combinations of first-, second-, and third-generation ALK inhibitors, based on the patient’s ALK mutation profile and the existing knowledge of the resistance or sensitivity of such mutations to different ALK inhibitors. The possibility of success of such strategy is suggested by a retrospective study of a cohort of 73 patients with ALK-positive NSCLC that received sequential therapy with different ALK inhibitors while enrolled in clinical trials [88]. In this study, sequential treatment with crizotinib followed by ceritinib led to a median combined PFS of 17.4 months, as compared to a median PFS of 8.2 months with crizotinib prior to the switch to ceritinib. More impressively, the OS for patients with metastatic ALK-positive lung cancer in this cohort exceeded 4 years from the time of metastasis diagnosis. Two patients that were poor responders to ceritinib had the ceritinib-resistance mutations C1156Y and 1151Tins, and the one patient with the ALK S1206Y mutation, previously shown to confer sensitivity to ceritinib, experienced a prolonged PFS of 14.8 months on ceritinib [88], supporting a relationship between the type of ALK mutation and patient response. Similar results from prospective studies will be key to inform the design of more effective patient-tailored protocols.

#### Combination therapy with other molecular targeted drugs

Various modalities of combination therapy are being considered in order to induce a durable response in patients who develop resistance to ALK inhibitors. Similar to the sequential ALK TKI strategy described above, this type of therapy would be personalized depending on repeated biopsies and determination of the specific resistance mechanism(s) that have evolved in the tumors [17]. Following are examples of promising combination therapies.

#### Combination therapy: EGFR inhibitors

A recent study indicates that there are at least three mechanisms by which EGFR activation can promote resistance to therapy targeting oncogenic kinase fusions in lung cancer, including those directed at ALK [89]. This would suggest, at least theoretically, that combined targeting of ALK and EGFR would be a more effective treatment for a patient exhibiting this specific resistance mechanism, compared to an ALK inhibitor alone. Indeed, as mentioned, ceritinib and alectinib were more effective in combination with the EGFR inhibitor afatinib when used to treat ceritinib- and alectinib-resistant NCI-H3122 cells with overactivation of EGFR pathways [77]. Two phase I clinical trials combining an ALK and an EGFR inhibitor have been reported to date, but neither of them involved patients with confirmed ALK mutation [3, 90]. What can be inferred from the dose reduction of crizotinib that was necessary in these trials, is that toxicity of combination therapy is a key issue to address in future clinical trials. In this regard, a dual ALK/EGFR inhibitor, called CHMFL-ALK/EGFR-050 (Compound 18), was recently developed [91]. CHMFL-ALK/EGFR-050 showed potent anti-tumor activity in pre-clinical NSCLC models driven by either mutant EGFR or ALK [91], but whether or not it will be suitable for NSCLC patients and a less toxic alternative for patients with dual ALK/EGFR overactivity, remains to be determined.

#### Combination therapy: heat shock protein 90 inhibitor

Heat shock protein 90 (HSP90) is thought to play a role in proper folding and stabilization of proteins, including those resulting from ALK fusions. Therefore, HSP90 inhibition leads to degradation of ALK fusion proteins, regardless of the ALK inhibitor-resistance mutations present [92]. Ganetespib, an inhibitor of HSP90, has been tested on NSCLC independently and in combination with crizotinib and other ALK inhibitors, showing improved anti-tumor effects both in vitro and in vivo, as compared to ALK inhibition alone [92]. Importantly, ganetespib overcame many forms of crizotinib resistance, including secondary ALK mutations commonly observed in patients [92].

The initial trial of ganetespib in NSCLC was a phase II study involving 99 patients with previously treated NSCLC and three molecular cohorts, including *EGFR*-mutated (*N* = 15), *KRAS* mutated (*N* = 17) and *EGFR/KRAS* wild type (*N* = 66). Ganetespib was administered at the recommended phase II dose of 200 mg/m^2^ intravenously on day 1, 8 and 15, in a 4-weekly schedule. The primary end point was PFS rate at 16 weeks. Only 4 patients in total had a partial response (PR), but when they were retrospectively tested for ALK rearrangement they were all ALK positive [93]. A phase I clinical trial (NCT01579994) evaluated ganetespib at 3 doses (100 mg/m^2^, 150 mg/m^2^ and 200 mg/m^2^) administered on day 1 and 8 of a 21-day cycle, in combination with crizotinib (250 mg twice daily, continuously) in twelve ALK-rearranged, ALK inhibitor-naïve patients with metastatic NSCLC. In this study, 67% (8/12) of patients had a PR and feasibility of the combination was demonstrated, warranting further trials [94]. However, follow-up trials comparing an ALK inhibitor alone to an ALK inhibitor plus ganetespib have not been reported by the time of completion of this review. Of interest, the GALAXY-2 phase III study that compared docetaxel plus ganetespib to docetaxel alone in advanced NSCLC, showed no benefit of adding ganetespib to chemotherapy [95]. For a comprehensive and up-to-date review of HSP90 and other HSP inhibitors in current clinical testing in NSCLC, see the recent article by Hendriks and Dingemans [96].

It is important to mention that a wide range of adverse effects are seen in patients treated with HSP90 inhibitors, partly due to their non-selective nature. These include diarrhea, nausea, vomiting, fatigue and retinal dysfunction leading to night blindness and blurred vision. More severe toxicities include grade III+ elevated hepatic enzymes, asthenia, and renal failure. In some cases, adverse effects led to discontinuation of treatment [93, 94, 96]. These toxicities highlight the need to maintain a good safety profile through dose limiting, especially when combining different treatments.

#### Cost-benefit of crizotinib treatment

Due to the cost of ALK inhibitors and the methods used to detect ALK-rearrangements, the cost-effectiveness of ALK targeted therapy has recently been brought into question. Djalalov et al. (2014) conducted a study on the cost-effectiveness of EML4-ALK diagnostic testing and first-line crizotinib therapy for patients with NSCLC from the Canadian Public Heath (Ontario) perspective [97]. They found that first-line crizotinib therapy provided patients with 0.379 additional quality-adjusted life-years (QALYs), but cost an additional $95,043 compared with standard care, and produced an incremental cost-effectiveness ratio of $250,632 per QALY gained. Mainly due to the cost of crizotinib, they determined that diagnostic testing and first-line treatment with crizotinib was not cost effective. Similar conclusions were reached by the same group regarding diagnostic testing in combination with crizotinib treatment as second line therapy for NSCLC patients eligible for chemotherapy [98]. Lower drug costs would be required to make ALK-targeting strategies economically feasible for both first- and second-line therapy. Nevertheless, it should be pointed out that the updated 2017 guidelines from The American Society of Clinical Oncology (ASCO) recommends crizotinib for first-line therapy of Stage IV NSCLC with a confirmed ALK rearrangement [99]. The greatest challenge for the treatment of ALK-rearranged NSCLC in the future, whether using sequential ALK inhibitors and/or combined therapies involving ALK and other inhibitors, is to significantly enhance QALYs while reducing costs.

## Conclusions

Upon discovery of aberrant ALK activity in lung cancer, the pharmaceutical industry was quick to develop effective targeted therapies that proved to be superior to chemotherapeutic regimens. In parallel, the development of ALK diagnostic tests to guide these therapies has also been rapidly progressing, yielding the standard approved methods widely used today, such as IHC and FISH, and others with high probability of prompt implementation due to improved sensitivity and specificity, such as qRT-PCR and NGS. Treatment with ALK inhibitors initially increased the progression-free survival of patients by an average of approximately 4 months, reduced severity of symptoms, and provided patients with an overall greater quality of life in comparison to chemotherapy. However, drug resistance is a major limiting factor, and the prognosis of patients with ALK-positive lung cancer is still less-than-optimal. Furthermore, ALK inhibitors such as crizotinib are expensive, and their cost-effectiveness is brought into question when they improve progression-free survival by just one-third of a year. Hopefully, future studies focused on combination therapy and other unique forms of treatment will uncover improved (and desirable cost-effective) treatment modalities for patients with ALK-positive NSCLC. Knowledge-based sequential treatment with first-, second- and third- generation ALK inhibitors is a promising strategy, while combination of ALK and other inhibitors is another option. A key aspect to keep in mind with combination therapies will be the potentially exacerbated toxicities and/or the emergence of unexpected toxicities.

## Abbreviations

AKT: AKR mouse thymoma
ALCL: Anaplastic large-cell lymphoma
ALK: Anaplastic lymphoma kinase
ASCO: American society of clinical oncology
Cas9: CRISPR-associated protein 9 nuclease
CCSP: Clara cell secretory protein
CLIP1: CAP-GLY domain-containing linker protein 1
CNS: Central nervous system
CRISPR: Clustered regularly interspaced short palindromic repeats
DCTN1: Dynactin subunit 1
EGFR: Epidermal growth factor receptor
EML4: Echinoderm microtubule-associated protein-like 4
ERK: Extracellular signal regulated kinase
FAM: Family with sequence similarity
FDA: Federal drug administration (United States)
FFPE: Formalin-fixed and paraffin-embedded
FISH: Fluorescent in situ hybridization
GCC2: GRIP and coiled-coil domain-containing protein 2
GRB2: Growth factor receptor-bound protein 2
HSP90: Heat shock protein 90
IHC: Immunohistochemistry
JAK: Janus kinase
KRAS: Kirsten viral rat sarcoma (v-RAS) homolog
MAPK: Mitogen activated protein kinase
NGS: Next generation sequencing
NPM: Nucleophosmin
NRG1: Neuregulin-1
NSCLC: Non-small cell lung carcinoma
ORR: Objective response rate
OS: Overall survival
PFS: Progression free survival
P-gp: P-glycoprotein
PI3K: Phosphatidylinositol-3-kinase
PLCγ: Phospholipase C gamma
PR: Partial response
QALYs: Quality adjusted life year
qRT-PCR: Quantitative reverse transcription polymerase chain reaction
RNAi: RNA interference
SHC1: Src homology 2 domain-containing transforming protein 1
SPC: Surfactant protein-c
TKI: Tyrosine kinase inhibitor
WHO: World Health Organization
